# Growth and Flowering Responses of Cut Chrysanthemum Grown under Restricted Root Volume to Irrigation Frequency

**DOI:** 10.1155/2014/254867

**Published:** 2014-11-16

**Authors:** Viyachai Taweesak, Thohirah Lee Abdullah, Siti Aishah Hassan, Nitty Hirawaty Kamarulzaman, Wan Abdullah Wan Yusoff

**Affiliations:** ^1^Department of Crop Science, Faculty of Agriculture, University Putra Malaysia, 43400 Serdang, Selangor, Malaysia; ^2^Department of Agribusiness and Information System, Faculty of Agriculture, University Putra Malaysia, 43400 Serdang, Selangor, Malaysia; ^3^Agro Technology Park, MARDI Cameron Highlands, Tanah Rata, 39007 Pahang, Malaysia

## Abstract

Influences of irrigation frequency on the growth and flowering of chrysanthemum grown under restricted root volume were tested. Chrysanthemum cuttings (*Chrysanthemum morifolium* “Reagan White”) were grown in seedling tray which contained coconut peat in volumes of 73 and 140 cm^3^. Plants were irrigated with drip irrigation at irrigation frequencies of 4 (266 mL), 6 (400 mL), and 8 (533 mL) times/day to observe their growth and flowering performances. There was interaction between irrigation frequency and substrate volume on plant height of chrysanthemum. Plants grown in 140 cm^3^ substrates and irrigated 6 times/day produced the tallest plant of 109.25 cm. Plants irrigated 6 and 8 times/day had significantly higher level of phosphorus content in their leaves than those plants irrigated 4 times/day. The total leaf area, number of internodes, leaf length, and leaf width of chrysanthemums grown in 140 cm^3^ substrate were significantly higher than those grown in 73 cm^3^ substrate. The numbers of flowers were affected by both irrigation frequencies and substrate volumes. Chrysanthemums irrigated 8 times/day had an average of 19.56 flowers while those irrigated 4 times/day had an average of 16.63 flowers. Increasing irrigation frequency can improve the growth and flowering of chrysanthemums in small substrate volumes.

## 1. Introduction

Many soilless systems have been studied for growing cut chrysanthemums. However, the production of chrysanthemum in soilless culture still has some problems to be resolved. Chrysanthemum grown in hydroponic systems was easily infected by* Pythium* [1, 2]. Growing them in substrate had greater possibility for commercial production such as in sand culture [3]. However, using a high amount of substrate will increase production costs for replacing the substrate. Reducing substrate volume can be a possible solution for production in substrate culture [4]. To date, there have been few studies on the growth and flowering response of cut chrysanthemums in substrates of limited volume.

Restricted root volume will limit water and nutrient availability. Many studies have reported that higher frequencies of irrigation can improve plant growth in such limited substrates [5, 6]. High irrigation frequency can maintain moisture content at the root zone of plants grown in restricted substrate volume [7]. Moreover, high irrigation frequency can improve the uptake of nutrients through the replenishment of nutrients in the root zone and improve transport of nutrients by mass flow [8]. Röber and Hafez [9] found that chrysanthemum grown in substrates with high moisture produced high weights of shoots and flowers.

Chrysanthemums that were grown in rockwool and given nutrients 1 or 3 times/day produced satisfactory growth [10]. In the case of nutrient film technique, vegetative growth of chrysanthemums in substrates of volume less than 100 mL can be maintained by fertigation 8 times/day [11]. Gisleröd [12] found that irrigation frequency for growing cut chrysanthemum in substrate depended on the season and variety. The optimal frequency of irrigation should be tested for different substrates [13]. This experiment investigated the effects of irrigation frequency on the growth and flowering of cut chrysanthemum grown in restricted root volumes.

## 2. Materials and Methods

The experiment was conducted from March, 2014, to June, 2014, at Agro Technology Park, Malaysia Agriculture and Development Institute, Pahang, Malaysia, in a shade house with an average temperature of 25.8°C and relative humidity of 70.50%. Rooted cuttings of* Chrysanthemum morifolium* “Reagan White” were grown in coconut peat at 73 and 140 cm^3^ volumes contained in seedling trays and a plant density of 64 plant/m^2^. Plants were irrigated with a nutrient solution by drip irrigation at frequencies of 4 (266 mL), 6 (400 mL), and 8 (533 mL) times/day. The nutrient solution used in the irrigation system from the first week to the seventh week included N 250, P 30, K 200, Ca 150, Mg 50, Fe 1.05, Mn 0.58, Zn 0.35, B 1, Cu 0.05, and Mo 0.05 mg/L. After the seventh week and until harvest, the nutrient solution contained N 200, P 30, K 200, Ca 150, Mg 50, Fe 1.05, Mn 0.58, Zn 0.35, B 1, Cu 0.05, and Mo 0.05 mg/L. The pH of the nutrient solution was maintained between 5.5 and 6.5 and the electric conductivity (EC) was 1.3–1.5 mS/cm. Night break was provided for 8 weeks from 11.00 pm. to 3.00 am.

Plant growth parameters such as plant height, stem diameter, leaf length, and leaf width were measured from two plant samples. Total leaf number and number of internodes were also counted. Root length was measured by using WinRHIZO, image analysis program (Regent instruments, Canada). Plant samples were dried in an oven at 70°C at least 48 hours, from which the dry weights of leaves, stems, roots, and flowers were determined. Leaf area was measured by Li-3100 area meter (LiCor Biosciences, USA). Leaf water potential was measured from fully expanded leaves by using a pressure chamber (Skye Instruments, UK) every two week and averaged means were used. Chlorophyll fluorescence was measured by Handy PEA (Hansatech Instruments, UK) to observe plant stress. Fully expanded leaf blades were darkened for 15 minutes by leaf clip. Sensor head was attached to the leaf clip, the shutter was opened and maximum quantum efficiency (Fv/Fm) was recorded.

Dry leaves at 14 weeks were ground and digested for nutrient analysis. Nitrogen (N) and phosphorus (P) were analyzed by autoanalyzer QuickChem 8000 (Lachat instruments, USA) and potassium (K), calcium (Ca), and magnesium (Mg) were analyzed using PerkinElmer 3110 atomic absorption spectrophotometer (PerkinElmer, USA).

At the point of harvesting, inflorescence diameter, number of flowers, number of petals, and flower diameter were observed. Lightness, chroma, and hue value of petal from three inflorescences were determined by CR-400 chroma meter (Konica Minolta, Japan). Stems of two flowers at a length of 35 cm were put in distilled water 300 mL at 25 ± 1°C, relative humidity 50–55%, and light intensity 1.3 *μ*mol m^−2^ s^−1^ for 10 hours per day to observe vase life.

Analysis of variance was calculated by SAS statistical software and means were compared by Tukey's test at *P* < 0.05.

## 3. Results and Discussion

**Figure 1:**
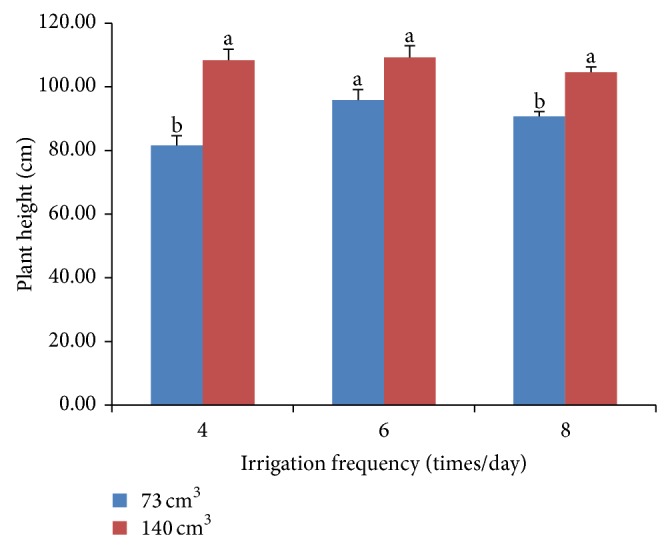
Interaction effects of irrigation frequencies and substrate volumes on plant height of chrysanthemum.

There were significant interactions between irrigation frequency and substrate volume on plant height of chrysanthemum grown in substrate culture. As shown in Figure 1, greater substrate volume resulted in taller plant as compared to lower substrate volume at irrigation frequencies 4 and 8 time/day. The tallest plant of 109.25 cm was obtained from chrysanthemum grown in 140 cm^3^ irrigated 6 times/day. This corresponded with previous result with marigold by Latimer [14] who reported that increasing container volume resulted in higher plant heights. However, plant heights of chrysanthemum grown in all substrate volumes were lower than those grown in soil (data not shown). This may be due to the stress conditions present for plants grown under restricted root volumes.

**Figure 2:**
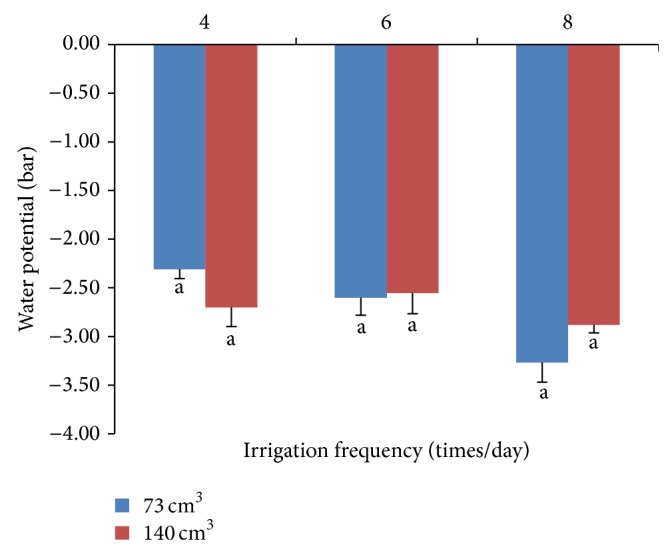
Interaction effects of irrigation frequencies and substrate volumes on water potential in leaves of chrysanthemum.

Chlorophyll fluorescence efficiency or Fv/Fm values were not significantly different between substrate volumes or irrigation frequencies (Tables 1 and 2). Chrysanthemums grown in all volumes and at all irrigation frequencies had average Fv/Fm values that were lower than the average value of 0.84 of normal plants [15]. This result confirms that the plants experienced some stress. Chlorophyll content did not differ significantly by either variable. Both substrate volumes and irrigation frequencies influenced the water potential in the leaves, but those differences were not significant (Figure 2).

**Figure 3:**
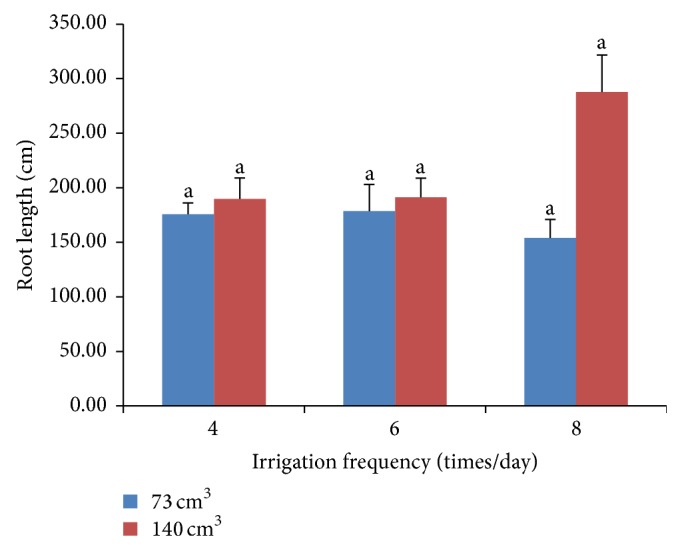
Interaction effects of substrate volumes and irrigation frequencies on root length of chrysanthemum.

The substrate volumes had pronounced effects on growth characteristics (Table 3). Even stem diameters did not differ significantly between two substrate volumes. Chrysanthemum grown in substrate volumes of 140 cm^3^ had a larger number of internodes. Furthermore, total leaf area and number of leaves correlated to substrate volume. Leaf area of plants grown in 140 cm^3^ was higher than that in 73 cm^3^ by 69%. This result was in agreement with other findings that found that increased substrate volume led to an increase in leaf area of chrysanthemum [11] and marigold [14]. Increasing substrate volume also increased leaf length and leaf width. Irrigation frequency, on the other hand, did not significantly affect shoot growth (Table 4). Differences between irrigation frequencies were not observed on stem diameter, number of internodes, leaf area, leaf number, leaf length, and leaf width. Substrate volume and irrigation frequency had an interaction effect on root length of chrysanthemum. However, there was no significant difference between each level of treatments alone but larger volumes tended to have longer root (Figure 3).

**Figure 4:**
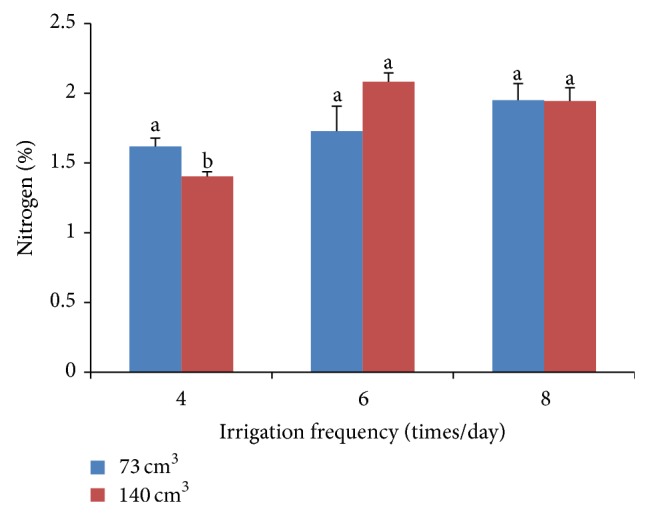
Interaction effects of substrate volumes and irrigation frequencies on nitrogen level of chrysanthemum.

There was an interactive relationship between substrate volumes and irrigation frequencies upon the levels of nitrogen in leaves. When irrigated 4 times/day, nitrogen levels in smaller substrate volumes were higher than those in larger substrate volumes (Figure 4). When irrigated 6 and 8 times/day, there was no significant difference between the substrate volumes.

Substrate volume did not show significant differences for levels of phosphorus, potassium, calcium, and magnesium in the leaves of chrysanthemum (Table 5). But phosphorus level was influenced by irrigation frequency. Chrysanthemum irrigated 6 and 8 times/day had significantly higher phosphorus levels than when irrigated 4 times/day (Table 6). This result indicates that higher irrigation frequencies can improve availability of phosphorus [16]. On the other hand, potassium, calcium, and magnesium levels were not influenced by irrigation frequencies.

Substrate volumes and irrigation frequencies influenced dry weight partitioning between plant parts. Increasing substrate volume increased leaf, stem root, flower, and total dry weight significantly as shown in Table 7. The increasing of root dry weight confirmed previous result by Goto et al. [11] who found that root dry weight of chrysanthemum increased with an increase of substrate volume. Total dry weight in 140 cm^3^ had total dry weight of 24.16 g, which was higher than in 73 cm^3^ by 47.13%.

Chrysanthemums irrigated 6 and 8 times/day had higher leaf dry weight, stem dry weight, and total dry weight than those irrigated 4 times/day as shown in Table 8. Schuch et al. [17] reported that chrysanthemums grown with high rates of irrigation produced higher stem dry weight than those obtained with lower irrigation frequency while root dry weight did not differ between irrigation frequencies. Chrysanthemum irrigated 8 times/day produced the highest flower dry weight. For total dry weight, plants irrigated 6 and 8 times/day were significantly higher than those irrigated 4 times/day by a factor of 32% and 23%. Katsoulas et al. [18] also founded that increasing irrigation frequency increased total dry mass of rose.

The number of flowers was affected by both substrate volumes and irrigation frequencies (Tables 9 and 10). When grown in 140 cm^3^, there were 33% more flowers than in 73 cm^3^. Irrigation 8 times/day produced the highest number of flowers, an average of 20.44. However, inflorescence diameter, flower diameter, number of petals, flower color, and vase life were not significantly influenced by either irrigation frequency or substrate volume. This outcome accorded with the studied one in gerbera, in which flower diameter was not influenced by irrigation frequency [19]. Carvalho et al. [20] noted that flower size of chrysanthemum was mainly influenced by genetic inheritance.

## 4. Conclusions

Results from this experiment demonstrated that irrigation frequencies of 6 and 8 times/day can improve plant growth and flowering characteristics such as plant dry weight and number of flowers of chrysanthemum plants grown under restricted root conditions. This may be attributable to a higher availability of nitrogen and phosphorus. We conclude that for cultivation efficiency, an irrigation frequency of 6 times/day can be suggested for growing chrysanthemums under restricted root volume.

However, further studies are required to confirm that increasing the irrigation frequency can improve plant growth under restricted conditions.

## Figures and Tables

**Table 1:** Effect of substrate volumes on chlorophyll fluorescence efficiency (Fv/Fm) and chlorophyll content.

Substrate volume (cm^3^)	73	140
Fv/Fm	0.68^a^	0.73^a^
Chlorophyll content	53.25^a^	53.99^a^

Values in each column accompanied by different letters differ significantly at *P* < 0.05 by Tukey's test.

**Table 2:** Effect of irrigation frequencies on chlorophyll fluorescence efficiency (Fv/Fm) and chlorophyll content.

Irrigation frequency (times/day)	4	6	8
Fv/Fm	0.70^a^	0.69^a^	0.73^a^
Chlorophyll content	53.21^a^	54.12^a^	53.53^a^

Values in each column accompanied by different letters differ significantly at *P* < 0.05 by Tukey's test.

**Table 3:** Effect of substrate volumes on shoot growth of chrysanthemum.

Substrate volume (cm^3^)	73	140
Stem diameter (cm)	0.57^a^	0.58^a^
Number of internodes	38.63^b^	44.75^a^
Leaf area (cm^2^)	487.69^b^	825.15^a^
Leaf number	67.33^b^	89.29^a^
Leaf length (cm)	9.22^b^	11.38^a^
Leaf width (cm)	5.21^b^	6.20^a^

Values in each column accompanied by different letters differ significantly at *P* < 0.05 by Tukey's test.

**Table 4:** Effect of irrigation frequencies on shoot growth of chrysanthemum.

Irrigation frequency (times/day)	4	6	8
Stem diameter (cm)	0.58^a^	0.58^a^	0.58^a^
Number of internodes	40.19^a^	44.25^a^	40.63^a^
Leaf area (cm^2^)	577.28^a^	693.84^a^	698.14^a^
Leaf number	67.69^a^	86.81^a^	80.44^a^
Leaf length (cm)	9.91^a^	10.73^a^	10.26^a^
Leaf width (cm)	5.56^a^	5.96^a^	5.59^a^

Values in each column accompanied by different letters differ significantly at *P* < 0.05 by Tukey's test.

**Table 5:** Effect of substrate volumes on phosphorus, potassium, calcium, and magnesium level in leaves of chrysanthemum at 14 weeks after transplanting.

Substrate volume (cm^3^)	73	140
P (%)	0.25^a^	0.25^a^
K (%)	4.43^a^	5.29^a^
Ca (%)	1.45^a^	1.58^a^
Mg (%)	0.23^a^	0.27^a^

Values in each column accompanied by different letters differ significantly at *P* < 0.05 by Tukey's test.

**Table 6:** Effect of irrigation frequencies on phosphorus, potassium, calcium, and magnesium level in leaves of chrysanthemum at 14 weeks after transplanting.

Irrigation frequency (times/day)	4	6	8
P (%)	0.14^b^	0.17^a^	0.17^a^
K (%)	2.68^a^	3.07^a^	3.16^a^
Ca (%)	1.18^a^	1.17^a^	1.22^a^
Mg (%)	0.25^a^	0.25^a^	0.26^a^

Values in each column accompanied by different letters differ significantly at *P* < 0.05 by Tukey's test.

**Table 7:** Effect of substrate volumes on leaf dry weight, stem dry weight, root dry weight, flower dry weight, and total dry weight.

Substrate volume (cm^3^)	73	140
Leaf (g)	3.44^b^	5.07^a^
Stem (g)	10.66^b^	15.60^a^
Root (g)	0.40^b^	0.67^a^
Flower (g)	1.92^b^	2.82^a^

Total (g)	16.42^b^	24.16^a^

Values in each column accompanied by different letters differ significantly at *P* < 0.05 by Tukey's test.

**Table 8:** Effect of irrigation frequencies on leaf dry weight, stem dry weight, root dry weight, flower dry weight, and total dry weight.

Irrigation frequency (times/day)	4	6	8
Leaf (g)	3.68^b^	4.85^a^	4.23^a^
Stem (g)	10.96^b^	14.91^a^	13.52^a^
Root (g)	0.50^a^	0.58^a^	0.53^a^
Flower (g)	2.02^b^	2.29^ab^	2.78^a^

Total (g)	17.16^b^	22.64^a^	21.07^a^

Values in each column accompanied by different letters differ significantly at *P* < 0.05 by Tukey's test.

**Table 9:** Effect of substrate volumes on flowering characteristics of chrysanthemum.

Substrate volume (cm^3^)	73	140
Inflorescence diameter (cm)	12.57^a^	13.52^a^
Number of flowers	16.21^b^	21.54^a^
Flower diameter (cm)	6.37^a^	6.06^a^
Number of petals	25.50^a^	25.35^a^
Flower color		
Lightness	75.92^a^	77.05^a^
Chroma	2.93^a^	2.96^a^
Hue	81.33^a^	83.84^a^
Vase life (days)	20.96^a^	21.21^a^

Values in each column accompanied by different letters differ significantly at *P* < 0.05 by Tukey's test.

**Table 10:** Effect of irrigation frequencies on flowering characteristics of chrysanthemum.

Irrigation frequency (times/day)	4	6	8
Inflorescence diameter (cm)	11.92^a^	13.59^a^	13.62^a^
Number of flowers	16.63^b^	19.56^ab^	20.44^a^
Flower diameter (cm)	5.71^a^	6.19^a^	6.76^a^
Number of petals	26.31^a^	25.33^a^	24.63^a^
Flower color			
Lightness	70.76^a^	81.40^a^	77.30^a^
Chroma	2.67^a^	3.17^a^	3.00^a^
Hue	78.76^a^	86.66^a^	82.33^a^
Vase life (days)	21.19^a^	20.81^a^	21.25^a^

Values in each column accompanied by different letters differ significantly at *P* < 0.05 by Tukey's test.

## References

[1] Liptay A., Tu J. C. (2003). Hydroponic chrysanthemum production: cultural and pathological issues. *Communications in Agricultural and Applied Biological Sciences*.

[2] Liu W., Sutton J. C., Grodzinski B., Kloepper J. W., Reddy M. S. (2007). Biological control of pythium root rot of chrysanthemum in small-scale hydroponic units. *Phytoparasitica*.

[3] Wilson D. P., Finlay A. R. (1995). Hydroponic system for the production of all-year-round chrysanthemum. *Acta Horticulturae*.

[4] Buwalda F., Baas R., van Weel P. A. (1986–1994). A soilless ebb-and-flow system for all year round chrysanthemums. *Acta Horticulturae*.

[5] Pires R. C. D. M., Furlani P. R., Ribeiro R. V., Bodine J. D., Sakai E., Lourenção A. L., Neto A. T. (2011). Irrigation frequency and substrate volume effects in the growth and yield of tomato plants under greenhouse conditions. *Scientia Agricola*.

[6] Scagel C. F., Bi G., Fuchigami L. H., Regan R. P. (2012). Irrigation frequency alters nutrient uptake in container-grown rhododendron plants grown with different rates of nitrogen. *HortScience*.

[7] Gizas G., Savvas D. (2007). Particle size and hydraulic properties of pumice affect growth and yield of greenhouse crops in soilless culture. *HortScience*.

[8] Silber A., Xu G., Levkovitch I., Soriano S., Bilu A., Wallach R. (2003). High fertigation frequency: the effects on uptake of nutrients, water and plant growth. *Plant and Soil*.

[9] Röber R., Hafez M. (1982). The influence of different water supply upon the growth of chrysanthemum. *Acta Horticulturae*.

[10] Paul V. N., William F. C. (1991). Physical analysis of rockwool slabs and effects of fiber orientation, irrigation frequency and propagation technique on chrysanthemum production. *Journal of Plant Nutrition*.

[11] Goto T., Takaya N., Yoshioka N., Yoshida Y., Kageyama Y., Konishi K. (2001). Effects of water and nutrient stresses on reduction of vegetative growth in chrysanthemum grown under restricted root zone volume. *Journal of the Japanese Society for Horticultural Science*.

[12] Gisleröd H. R. (1988). Effects of watering frequency on growth of cut chrysanthemums. *Acta Horticulturae*.

[13] Raviv M., Wallach R., Silber A., Medina S., Krasnovsky A. (1999). The effect of hydraulic characteristics of volcanic materials on yield of roses grown in soilless culture. *Journal of the American Society for Horticultural Science*.

[14] Latimer J. G. (1991). Container size and shape influence growth and landscape performance of marigold seedlings. *HortScience*.

[15] Maxwell K., Johnson G. N. (2000). Chlorophyll fluorescence—a practical guide. *Journal of Experimental Botany*.

[16] Xu G., Levkovitch I., Soriano S., Wallach R., Silber A. (2004). Integrated effect of irrigation frequency and phosphorus level on lettuce: P uptake, root growth and yield. *Plant and Soil*.

[17] Schuch U. K., Redak R. A., Bethke J. A. (1998). Cultivar, fertilizer, and irrigation affect vegetative growth and susceptibility of chrysanthemum to western flower thrips. *Journal of the American Society for Horticultural Science*.

[18] Katsoulas N., Kittas C., Dimokas G., Lykas C. (2006). Effect of irrigation frequency on rose flower production and quality. *Biosystems Engineering*.

[19] Tsirogiannis I., Katsoulas N., Kittas C. (2010). Effect of irrigation scheduling on gerbera flower yield and quality. *HortScience*.

[20] Carvalho S. M. P., Heuvelink E., Harbinson J., van Kooten O. (2006). Role of sink-source relationships in chrysanthemum flower size and total biomass production. *Physiologia Plantarum*.

